# Compartment syndrome and suicide attempt

**DOI:** 10.1192/j.eurpsy.2021.1719

**Published:** 2021-08-13

**Authors:** E. Rodríguez Vázquez, C. Capella Meseguer, J. Gonçalves Cerejeira, I. Santos Carrasco, M. Queipo De Llano De La Viuda, A. Gonzaga Ramírez, G. Guerra Valera

**Affiliations:** 1 Psiquiatría, Hospital Clínico Universitario de Valladolid, Valladolid, Spain; 2 Psiquiatría, HCUV, Valladolid, Spain

**Keywords:** liaison psychiatry, Suicide Attempt, compartment syndrome

## Abstract

**Introduction:**

The compartment syndrome is a pathological condition characterized by a decrease, or even interruption, of the microcirculation within a soft tissue compartment. There have been a few cases reported about compartment syndrome due to a suicide attempt.

**Objectives:**

To present an unusual complication of an autolytic attempt

**Methods:**

A descriptive study of a clinical case and literature review

**Results:**

A 49-year-old woman, divorced. With no psychiatric history and no somatic antecedents. Comes to the hospital after been found lying face down on the bathroom’s floor for 48 hours, next to her two empty blister packs of lorazepam and naproxen. Her partner says they argued two days ago. Brain CT: with no abnormalities. Blood analysis: metabolic acidosis with rhabdomyolysis and kidney failure. She presents ischemic injuries in both inferior extremities with right food ischemia and with no pedal pulses. Compartment syndrome is diagnosed, being necessary a bilateral fasciotomy and later a right lower extremity amputation. Initiates referral from Vascular Surgery for self-poisoning. She refers to low mood and mild anxiety due to work and relationship issues/problems. She accepts that she self-poisoning only to attract her partner’s attention after the argument. The examination shows logical thought, emotional lability, good judgement, future-oriented without suicidal ideation. Clinical judgement: acute stress reaction.

**Conclusions:**

The compartment syndrome is a rare complication of the suicide attempt. Our patient suffered a compartment syndrome lying on the bathroom’s floor for 48 consecutive hours without apparent trauma and no somatic antecedents. This syndrome could be developed by high naproxen and lorazepam intake.

**Disclosure:**

No significant relationships.

